# Epigenetics in Sepsis: Understanding Its Role in Endothelial Dysfunction, Immunosuppression, and Potential Therapeutics

**DOI:** 10.3389/fimmu.2019.01363

**Published:** 2019-06-18

**Authors:** Deborah Cross, Ruth Drury, Jennifer Hill, Andrew J. Pollard

**Affiliations:** Oxford Vaccine Group, Department of Paediatrics, NIHR Oxford Biomedical Research Centre, University of Oxford, Oxford, United Kingdom

**Keywords:** sepsis, epigenetics, immunosuppression, endothelial dysfunction, histone deacetylase inhibitors

## Abstract

Sepsis has a complex pathophysiology in which both excessive and refractory inflammatory responses are hallmark features. Pro-inflammatory cytokine responses during the early stages are responsible for significant endothelial dysfunction, loss of endothelial integrity, and organ failure. In addition, it is now well-established that a substantial number of sepsis survivors experience ongoing immunological derangement and immunosuppression following a septic episode. The underpinning mechanisms of these phenomena are incompletely understood yet they contribute to a significant proportion of sepsis-associated mortality. Epigenetic mechanisms including DNA methylation, histone modifications, and non-coding RNAs, have an increasingly clear role in modulating inflammatory and other immunological processes. Recent evidence suggests epigenetic mechanisms are extensively perturbed as sepsis progresses, and particularly play a role in endothelial dysfunction and immunosuppression. Whilst therapeutic modulation of the epigenome is still in its infancy, there is substantial evidence from animal models that this approach could reap benefits. In this review, we summarize research elucidating the role of these mechanisms in several aspects of sepsis pathophysiology including tissue injury and immunosuppression. We also evaluate pre-clinical evidence for the use of “epi-therapies” in the treatment of poly-microbial sepsis.

## Introduction

### An Overview of Sepsis Pathophysiology

Sepsis is a syndrome with a broad clinical manifestation, defined by The Third International Consensus Definitions for Sepsis and Septic Shock (Sepsis-3) as “a life-threatening organ dysfunction caused by dysregulated host responses to infection” (1). Due to the numerous possible presentations, sepsis can be a difficult clinical condition to recognize, especially during the early stages if patients exhibit non-specific symptoms of being unwell (1–4) or if archetypal signs of infection are absent, e.g., in young infants, the elderly, and the immunocompromised (5–8). Signs which are highly suggestive of sepsis include (but are not limited to) acute confusion, hypotension, tachycardia, and tachypnoea, hypoxia, reduced urine production, a high blood lactate level and a non-blanching rash. Only one of these signs may be present, and none are unique to sepsis (9).

Screening tools have been developed to aid identification of patients who are seriously ill with suspected sepsis (10–12). An example of one such tool, the Sequential Organ Failure Assessment (SOFA) score, codifies the progression of sepsis-related organ failure (13). However, despite these efforts to improve diagnostics, sepsis still can be missed, leading to delays in treatment which can dramatically worsen outcomes. Rapid administration of antibiotics is critical; for every hour of delayed treatment, mortality risk increases by 7.6% (14). The development of tests that accurately predict the onset of sepsis before organ failure occurs would be useful for improving outcomes. The broad manifestation and rapid onset of sepsis make this very challenging, but it nevertheless continues to be an active area of research (15–22).

Infection-driven inflammation causes substantial tissue injury and organ dysfunction during acute sepsis and represents a major cause of mortality (22, 23). The binding of commonly expressed, conserved pathogen antigens (pathogen-associated molecular patterns, PAMPs) to pattern recognition receptors activates NF-κB signaling and promotes transcription of a wide range of pro-inflammatory factors (24–26). The endothelium becomes activated, increasing its permeability as well as the adherence and migration of leucocytes (27). Loss of endothelial integrity drives intravascular leak, hypotension, and widespread oedema (27, 28). The production of damage-associated molecular patterns (DAMPs) from host cells feeds the inflammatory response, resulting in more tissue injury, and thereby, creating a vicious circle. Concurrent to this, cytokines with anti-inflammatory properties are produced in efforts to promote resolution of inflammation and tissue repair; antigen presenting cells become less responsive to lipopolysaccharide (LPS) and other PAMPs, widespread apoptosis of leucocytes is observed, and myeloid-derived suppressor cells (MDSCs) are substantially increased (9, 29). It was once thought that acute hyperinflammatory responses preceded an immunosuppressive phase, however, it is now believed that there two phases can exist simultaneously (30).

Individuals who clear infection can still exhibit protracted, deranged immune responses following a septic episode. Persistent inflammation, immunosuppression, and catabolism syndrome (PICS), whilst not a universal phenomenon in sepsis, describes a clinical syndrome that patients with longer ICU stays can exhibit (31, 32). One characteristic of PICS is increased susceptibility to opportunistic infections and reactivation of latent viruses, which contributes to morbidity and mortality after the initial infective insult has resolved (33, 34). The factors which contribute to PICS are multi-factorial but the significant risk of rehospitalization with infection may suggest an ongoing perturbation of the immune response (35). Indeed, a study by Arens et al. demonstrated persistent immunoparalysis weeks to years after sepsis (36). There is a paucity of studies which investigate the persistence of PICS after hospital discharge, however, it is notable that sepsis survivors have a significantly reduced survival rate over the years following acute infection vs. age matched individuals, occurring independently of health status preceding the septic episode (37–39). The increased death rate may result from persisting sequalae of sepsis; e.g., increased frailty, irreversible impairment in organ function and/or from sepsis-associated, sustained changes in immune function e.g., immunosuppression. Work is underway to elucidate the mechanisms behind these modifications, with some studies suggesting they arise from changes to the epigenome of leucocytes.

### Epigenetics: Definition and Mechanisms

**Figure 1:**
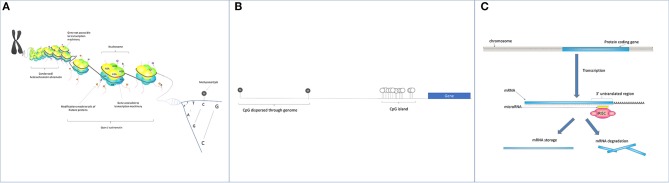
**(A)** Histone modifications: The negative charge of DNA allows it to bind tightly to positively charged histone proteins. DNA wraps around octomers of histone proteins and forms discrete units known as nucleosomes, the basis of chromatin. The overall structure and openness of chromatin is dictated by chemical modifications of the N terminal amino acid tails of the histone proteins. Chemical modifications include acetylation, methylation, phosphorylation, SUMOylation, citrullination, and ADP-ribosylation. **(B)** CpG methylation: The majority of cytosines found in cytosine-guanine dinucleotides (gray circles) are methylated. CpG-rich sections of the genome (CpG islands) occurs in areas requiring transcriptional control e.g., retrotransposons and gene promotors. Here, methylation status is more dynamic, with some hypomethylated CpGs (white circles) facilitating promotor accessibility and gene transcription. **(C)** Small non-coding RNAs interact with complementary sequences in DNA and on mRNA to interfere with gene transcription and translation respectively. A well-known species of small RNAs are microRNAs. Mature single stranded microRNAs molecules (21–24 nt long) are incorporated into the RNA induced silencing complex (RISC) and then bind to a complementary sequence in the 3'UTRs of mRNA molecules. This binding inhibits mRNA translation and results in either mRNA degradation or storage.

Epigenetics refers to the regulation of gene expression not caused by underlying changes in DNA sequence (40). In eukaryotes, DNA forms a stable structure with octomers of histone proteins; this stable structure is known as chromatin [Figure 1, (41)]. The “openness” of chromatin structure affects the accessibility of DNA to transcription factors and RNA polymerase II, and is therefore a key factor in determining the rate of mRNA expression (42). Three major epigenetic mechanisms are described, two of which exert their effect by influencing chromatin compaction (see Figure 1). DNA methylation is a modification of cytosine residues mainly in the context of cytosine-guanine (CpG) motifs. The majority of the mammalian genome is CpG poor, with enriched regions occurring at transcriptional regulatory loci such as promotors and enhancers (termed CpG islands). Around 60–70% of promotors contain CpG islands (43). The second mechanism is histone modification; post-translational modifications of the amino acids in the tail region of histone proteins which include acetylation, phosphorylation, ubiquitylation, and methylation. Modifications of amino acids at specific locations in the protruding tails either strengthen or weaken the interaction between DNA and histones. The final mechanism involves non-coding RNAs (ncRNAs), which can modulate gene expression by binding to either sites in the genome to prevent gene transcription or mRNA transcripts to prevent translation (44, 45).

There is growing evidence that modifications of the epigenome impacts the phenotype of immune cells in such a way as to affect responses to infection, and are involved in propagating inflammatory disorders (46, 47). Whilst most epigenetic marks are generally stable over time, those at certain loci show high plasticity in response to environmental factors such as smoking, diet, and disease, making them of interest in the context of various pathologies (48–50). The rewritable nature of epigenetic modifications and the responsiveness of epigenetic enzymes to inhibitor therapy creates great potential for this avenue of treatment in patients both with chronic and acute inflammatory diseases such as sepsis.

In this review, the epigenetic modifications associated with various stages of sepsis will be discussed. Specifically, we cover mechanisms involved in endothelial dysfunction during the hyperinflammatory response and those underpinning aspects of immunosuppression in PICS. The pre-clinical evidence for use of epi-therapies will also be described.

## Search Strategy

References were identified through Ovid using search terms (“sepsis” OR “septic shock” OR “endotoxin tolerance (ET)”) AND (“epigenomics” OR “epigenetic” OR “DNA methylation” OR “Histone modifications” OR “histone” OR “non-coding RNA” OR “micro RNA”). Bibliographies of papers of interest were searched by hand to identify additional studies. Relevant papers identified in the database were included.

## Epigenetic Changes Associated With Sepsis Pathophysiology

### Histone Acetyltransferases (HATs) and Histone Deacetylases (HDACs) as Regulators of Inflammation

Histone acetylation is a key process involved in regulating inflammatory response genes (51, 52). Addition or removal of acetyl groups is mediated by two families of antagonistic enzymes, histone acetyltransferases (HATs), and histone deacetylases (HDACs). There are numerous studies that associate levels of histone acetylation with expression of pro-inflammatory cytokines and other anti-microbial products (52). Therefore, understanding the relative activities of these two enzymatic groups has great relevance to sepsis. To date, five families of HATs enzymes have been discovered. Using acetyl-CoA as a substrate, these enzymes target primarily lysine residues on histones 3 and 4 (53). In humans, 18 HDACs have been discovered, grouped into four classes based on sequence homology with their yeast counterparts. Classes I, II, and IV represent the “classical” HDACs and are the most extensively studied. Class III HDACs, otherwise known as sirtuins, utilize a distinct mechanism for lysine deacetylation requiring NAD+ as a substrate, in contrast with classical HDACs which are Zn^2+^-dependent metalloproteases (54).

Whilst acetylation is generally considered a pro-transcriptional modification, increasing evidence suggests this is an over-simplistic view and that the effect of acetylation on chromatin structure is in fact site-specific (55, 56). Therefore, the roles of these enzymes in transcriptional regulation is likely to be highly complex and requires detailed elucidation as they are pursued as targets of therapeutics. In addition to histones, HATs, and HDACs have multiple non-histone targets that are critical for a range of cellular processes including metabolism and cell cycle (54).

### Epigenetic Changes Associated With Endothelial Dysfunction, Tissue Injury, and Organ Failure in Sepsis

Endothelial damage, as a result of an excessive cytokine response, is one of the initiating steps that ultimately leads to sepsis-associated organ dysfunction. Other than provision of fluids and use of inotropic drugs, there are no interventions available to restore loss in arterial partial pressure and organ perfusion (57). During sepsis, the endothelium is activated and adhesion molecules including ICAM, VCAM, and E-selectin are upregulated (58, 59). These adhesion molecules are critical for leucocyte infiltration into tissues. Entry of neutrophils into the endothelium in particular has been paradoxically associated with both containment of infection and exacerbation of tissue injury (60). Besides adhesion molecule upregulation, endothelial cell junctions become “loose,” leading to an increase in permeability and a loss of fluid from the vascular system into the surrounding tissues. Whilst neither of these processes are pathological in themselves, the extent to which they occur in sepsis is a major driver of organ failure. Therefore, stabilizing endothelial disruption could be an effective avenue of therapeutic intervention in sepsis.

Loss of histone acetylation during acute lung injury may partially drive the over-expression of adhesion molecules and regulate endothelial permeability. Acetylation loss at the promotors of *Angp1, Tek*, and *Kdr–*genes with critical roles in both Tie2/Angiopoietin and vascular endothelial growth factor (VEGF/VEGFR) signaling cascades–was observed in lung and extra-pulmonary organs in a mouse sepsis model (61). Loss of acetylation was suggested to be responsible for a significant reduction in gene expression 6 h post-induction of sepsis and for increased albumin leak. Despite the limitations of this study [as discussed in detail by Bataille et al. (62)], these findings highlight a potential mechanism by which inflammatory factors can influence epigenetic regulation and drive maladaptive changes in endothelium. Indeed, ICAM-1, and E-selectin expression are markedly reduced in the lungs of mice with poly-microbial sepsis if they are pretreated with histone deacetylase inhibitors (HDACi) (63, 64). Neutrophil infiltration and albumin leak were both minimized and associated with improved survival.

Other epigenetic mechanisms are also implicated in mediating tissue injury [extensively reviewed in (44)]. Perturbation of several micro RNAs (miRNAs) during sepsis has been described in plasma and the endothelium (65). MiR-181b expression in endothelial cells minimizes leucocyte invasion of tissues by reducing expression of adhesion molecules, mediated by suppression of NF-κB signaling (66). Injection of miR-181b mimics in a mouse model of endotoxemia downregulated VCAM-1 in the lung and reduced leucocyte adhesion and lung histopathology scores (66). Interestingly, 24 intensive care patients with sepsis have lower circulating levels of miR-181b than those with other inflammatory conditions, suggesting this mechanism is particularly pertinent in driving sepsis-associated overexpression of adhesion molecules (66). Suppression of NF-κB signaling by miRNAs may also confer protective effects in other organs. Animal models of sepsis-associated cardiac dysfunction have shown that miR-146a expression can attenuate NF-κB activation and inflammatory responses in both the myocardium and peripheral blood, changes associated with improved survival (67–69). Further mechanistic studies would be helpful to fully elucidate the functions of these miRNAs, and beyond this should explore whether their therapeutic modulation would be of benefit in sepsis.

### Persistent Inflammation, Immunosuppression, and Catabolism Syndrome (PICS)

Immunosuppression in critically ill patients was first noted in 1970's when it was discovered that these patients did not develop delayed hypersensitivity responses to common antigens (70). It is now recognized that ongoing immunological disturbance following sepsis occurs in a subset of patients, keeping them in intensive care with a milieu of symptoms despite clearance of initiating infection. These symptoms are collectively referred to as PICS. Individuals may exhibit inappropriately elevated protein catabolism (leading to loss of lean body mass and thus increased frailty), poor wound healing, an increased susceptibility to infection, and prolonged immunosuppression. Current prognosis is poor with many requiring extensive stays in intensive care and high rates of mortality (71). Understanding of the mechanistic features that drive immunosuppression is essential and likely to involve epigenetic elements.

#### Expansion of Myeloid-Derived Suppressor Cells (MDSCs)

Several studies have highlighted the extensive apoptosis of immune cells during acute sepsis as a prominent driver of subsequent immune dysfunction. Besides a depletion in sheer numbers of cells, several studies have noted functional abnormalities in the remaining subsets of the immune system. The proportional number of regulatory T-cells (Tregs) and other immunosuppressor subsets is significantly increased in patients with PICS. MDSCs are a subset of immature myeloid cells with highly immunosuppressive properties [reviewed extensively by Schrijver et al. (72)]. Specifically, their production of arginase-1, reactive oxygen species, TGF-β, and IL-10 critically suppress T-cell and NK cell function (73). These cells are largely absent in healthy individuals but form a major component of tumor micro-environments in cancer and are detectable in blood following sepsis (74). MDSCs associate with deleterious outcomes and are highly elevated in patients with PICS. Their considerable expansion following sepsis and function as immunosuppressors make understanding of their development an important area of research.

Epigenetic modulation of myeloid progenitors may explain the disproportional expansion in MDSCs. Transcriptional regulators in these cells are precisely controlled by numerous epigenetic mechanisms. Regulation of nuclear factor 1A (NFI-A) has been shown to be critical for myeloid cell differentiation (75). MiR-181b and miR-21, through a synergistic mechanism, negatively modulate NFI-A expression in mice subjected to cecal ligation and puncture (76). In this study, miR-181b and miR-21 were both upregulated in bone marrow, and blockade of these miRNAs substantially impeded MDSC expansion, improved the capacity of these mice to clear peritoneal infection, and increased survival. Other transcription factors, including C/EBP-β and Runx1, are also epigenetically regulated and drive MDSC expansion. HDAC11 is recruited to the C/EBP- β promoter and negatively controls its expression; in knockout models, loss of HDAC11 significantly increases MDSC populations (77). Whether HDAC11 is upregulated during sepsis is unclear. MDSC differentiation and suppressive capacity can also be altered by regulation of miR-9 which in turn exerts its effect by regulation of Runx1 (78). Elevation of miR-9 expression during sepsis is not confirmed, however it should be noted that it is inducible by LPS and several pro-inflammatory cytokines (79), making a strong case for activity during sepsis. Whether targeting the mechanisms that drive MDSC development could be of therapeutic benefit in sepsis is an unanswered question. The miRNAs and acetylation enzymes highlighted here have multiple targets and their inhibition may have other undesirable effects. Furthermore, specific targeting of regulatory mechanisms in these cells alone may prove a challenge. However, further exploration of this area is undoubtedly warranted.

#### Endotoxin Tolerance as a Mechanism Mediating Immunosuppression

The second notable aspect of immunosuppression is the hypo-responsiveness, particularly of innate immune cells, to subsequent challenge. ET is a well-described clinical phenomenon whereby pro-inflammatory responses to LPS are repressed during secondary encounter. Not all refractory responses are necessarily harmful–acute modulation of pro-inflammatory responses may in fact be beneficial during early sepsis. However, protracted suppression has detrimental consequences, potentially making patients more vulnerable to Gram-negative infections. Several epigenetic mechanisms have been linked to the persistence of ET (80, 81). Elevated miR-221 and miR-222 following prolonged LPS exposure were recently found to have a role in regulating Brahma-related gene 1, which in turn mediated transcriptional silencing of several pro-inflammatory products (80). In addition, failure to induce pro-transcriptional histone modifications–namely, acetylation and tri-methylation of histone 3– was shown by Foster et al. to repress pro-inflammatory gene expression on secondary LPS encounter, whilst leaving anti-microbial, and metabolic gene expression intact (81). Other studies have also demonstrated alterations in promotor histone profiles during sepsis–including loss of marks of active transcription–downregulating genes involved in pro-inflammatory responses and antigen presentation (82).

Some of the epigenetic machinery responsible for modifying histones has been demonstrated to be directly regulated by LPS [reviewed in (9)], potentially providing a mechanistic link between epigenetics and immune regulation. Expression of histone demethylase enzyme, JMJD3, was shown to be induced by LPS stimulation via NF-κB signaling in macrophages (83, 84). Furthermore, the activity of HAT and HDAC enzymes can also be modulated by LPS, although the extent to which their activity contributes to ET remains unclear. CREB-binding protein (CBP), a transcriptional co-activator with HAT activity, is critically involved in NF-κB signaling and regulation of the inflammatory responses (85). LPS exposure increases CBP stability by stimulating the removal of ubiquitin and blocking proteosomal degradation (86). This in turn correlates with increased histone acetylation and cytokine release. Stabilization of histone acetyltransferase HBO1 via a similar mechanism has also been reported (87). Conversely, sirtuins (class III HDAC) have demonstrable suppressive roles in cytokine regulation. Sirtuin 1 (SIRT1) rapidly accumulates at the proximal promotors of TNFα and IL1B following LPS stimulation and induces facultative heterochromatin formation thus silencing gene expression (88). In the same study, SIRT1 was additionally shown to deacetylate (and deactivate) the transcription factor NF-κB p65, a critical inducer of inflammatory signaling, preventing further transcription of pro-inflammatory genes. Another prominent sirtuin family member, SIRT6, can also act as an inflammatory repressor by deacetylating histone 3 at lysine 9 (H3K9) and inducing heterochromatin formation at NF-κB target gene promoters (89). In addition to acetylation changes, methylation and the enzymes which regulate methylation state are observed to negatively regulate expression of some of pro-inflammatory gene loci such as TNFα during sepsis immunosuppression (90).

Cytokines, TNF-α and type I interferons, have also been shown to modulate monocyte responsiveness to LPS through changes in the epigenome. Pre-treatment of monocytes *in vitro* with TNF-α prior to LPS stimulation was shown to block accumulation of euchromatin-associated H4ac and H3K4me3 at promotor regions of NF- κB target genes (90). When stimulated with LPS pre-treated monocytes had significantly lower pro-inflammatory mRNA expression than those without prior TNF-α exposure. Conversely, type I interferons propagated LPS responses by priming chromatin to respond, heightening sensitivity to weak upstream signaling. The ability of the immune response to self-modulate may represent a beneficial protective mechanism in the short-term. It is the timing and extent of the immunosuppression, specifically an inappropriate continuation once infection has cleared, which generates harm. It is notable that in most of these studies a very small selection of cytokines have been investigated (typically TNF-α and IL-6). Furthermore, no studies have characterized the persistence of epigenetic modifications, for example, in re-hospitalized patients after sepsis. Therefore, the ability of these described changes to potentiate long term suppression is difficult to assess. Repression of pro-inflammatory cytokines represents only one element of immunosuppression. Therefore, the contribution of epigenetic modulation of immune function to overall patient outcome remains to be fully elucidated.

### Additional Epigenetic States of Potential Relevance to Sepsis

Other epigenetic states have been associated with modulation of immune function and may be pertinent to inflammatory disorders such as sepsis. Trained immunity in innate cells was reported in 2011 by Netea et al. (91), defined as a heightened immune response to secondary challenge following sub-lethal exposure to an initial stimulus. This phenomenon was subsequently linked to deposition of permissive histone modifications, H3K4me3, at promotors of *tnf*α, *il6*, and *tlr4* in monocytes following antigen exposure (92). Primed monocytes were found to mount a stronger pro-inflammatory cytokine response during secondary challenge. Priming with other antigens such as fungal β-glucan has also been shown to increase H3K4me3 occupancy at pro-inflammatory gene promotors and correlate with increased cytokine release (93).

The induction of ET or trained immunity appears to be dependent on the microbial stimulus itself and antigen concentration (94). Stimulation of monocytes via Nod Like Receptor (NLR) or Toll Like Receptor (TLR) pathways resulted in unique effector functions, epigenetic and metabolic profiles (95, 96). Whilst TLR stimulation via LPS induced strongly immunosuppressive effects, NLR engagement had the opposite effect, enhancing effector function in a dose-dependent manner. Interestingly, tolerized monocytes regain responsiveness when stimulated with β-glucan (97). These findings underline the complexity of proposed innate immunological “memory.” A plethora of factors including the host cytokine milieu, the antigen in question and antigen concentration all influence the development of either refractory or enhanced effector function. In a complex immune response such as during sepsis, it is likely that a combination of these features occurs simultaneously. Which factors, epigenetic or otherwise, contribute to persistence of ET still require complete elucidation. That immune states such as trained immunity have been shown to propagate via progenitor cells suggests that alteration of host epigenetic regulation can persist extensively (98, 99). Therefore, characterization of histone alterations in sepsis survivors over a prolonged period of time would provide useful information on the longevity of sepsis-induced changes.

## Epigenetic Therapeutics: Potential and Limitations in Treatment of Sepsis-Associated Tissue Injury

### Histone Deacetylase Inhibitors (HDACi)

A significant amount of research has examined the effect of modulating epigenetic enzymes upon sepsis-associated organ dysfunction and outcome. Numerous histone deacetylase inhibitor studies in pre-clinical models of sepsis have been conducted (summarized in Table 1), discussed in detail in this section. Characterizing the effect of HDACi at the tissue level is difficult in humans. Animal models circumvent this limitation and have brought valuable insights.

**Table 1:** Summary of pre-clinical studies investigating the therapeutic potential of various HDACi inhibitors.

**Inhibitor**	**Target**	**Study**	**HDACi dose**	**Sepsis model**	**Animal model**	**Effect**
Valproic acid (VPA)	HDAC1 HDAC2	(100)	50 mg/kg	LPS	Beagles	Significant reduction in TNF-α and IL-6 mRNA in PBMCs 3 and 6 h post-treatment. No difference in clinical symptoms between treated and untreated groups.
		(101)	300 mg/kg	CLP	C57Bl/6J mice	Reduced TNF-α, IL-1β, and IL-6 in peripheral blood, reduced histopathological events, and oxidative damage in renal tissue. No comment on survival.
		(102)	Prophylactic and therapeutic doses given.	CLP	Sprague-Dawley rats	Improves survival of treated mice, inhibits transcription of TNF-α and IL-6, reduced oxidative burst. Reduced acute lung injury All animals were exposed to haemorrhagic shock 24 h prior to CLP.
		(103)	Prophylactic and therapeutic doses given.	CLP	BALB/c mice	Anti-apoptotic effect in lung and spleen tissue. No effect on serum cytokine levels or inflammation in lungs. No controls used.
		(104)	100 mg/kg	CLP	C57BL/6 mice	No significant difference in survival between treated and untreated mice. Hippocampal IL-1β levels were reduced in VPA group, Spatial learning ameliorated in treated mice.
Tubastatin A (Tub-A)	HDAC6	(105)	70 mg/kg	CLP	C57Bl/6J mice	Improved survival, inhibits transcription of TNF-α and IL-6, reduced oxidative burst. All animals were exposed to haemorrhagic shock 24 h prior to CLP.
		(106)	70 mg/kg	CLP	C57BL/6J mice	Improved survival, reduced TNF-α and IL-6 in peritoneal fluid and plasma, reduced lung injury, and macrophage apoptosis.
Trichostatin A (TSA)	HDAC1 HDAC2	(107)	1 mg/g (co-administered with DNA methyltransferase inhibitor, Aza)	LPS	C57BL/6J mice	Reduced apoptosis in lung tissue, reduced pro-apoptotic gene expression in lung.
		(108)	1 μg/g (administered alone or DNA methyltransferase inhibitor, Aza)	LPS	C57BL/6J mice	Treatment with both epigenetic modifiers had synergistic effect. Increased M2 macrophages in lungs, reduced pro-inflammatory cytokines in plasma, increased acetylation of STAT3 promotor in BMDMs, increased STAT3–Bcl2 signaling, reduced p38MAPK activation.
		(109)	3.3 μmol/L/kg (administered alone or DNA methyltransferase inhibitor, Aza) Co-administered with 4.4 μmol/L/kg Aza	LPS	C57BL/6 mice	Treatment with both epigenetic modifiers had synergistic effect. Reduced inflammation in lung Reduced pulmonary microvascular permeability Reduced apoptosis of lung cells
		(110)	10 mg/kg	CLP	Male Sprague–Dawley rats	Daily treatment for 7 days reduced neuronal cell death and improved spatial learning and memory defects induced by sepsis.
		(111)	10 mg/kg	CLP	Sprague-Dawley rats	HDAC inhibition increased skeletal muscle catabolism 4 h after sepsis induction, atrogin-1 expression is upregulated.
Suberoylanilide hydroxamic acid (SAHA or Vorinostat)	Pan-inhibitor	(112)	50 mg/kg	CLP	C57BL/6J mice	Improved survival, ameliorated coagulation disturbances at 48 h post-sepsis induction.
		(113)	50 mg/kg	CLP	C57BL/6J mice	Improved survival, reduced cytokine levels in peritoneal fluid and blood, reduced acute liver injury.
		(114)	Prophylactic and therapeutic doses given.	LPS	C57B1/6J mice	Reduced phosphorylation of MAP kinase proteins at 3 h post-induction, reduced neutrophil, and macrophage activity in the liver, reduced pro-inflammatory cytokine levels in liver tissue.
		(115)	50 mg/kg	LPS	C57BL/6J mice	Improved survival, reduced MyD88 gene expression, reduced TNF-α and IL-6 production.
		(116)	Prophylactic and therapeutic doses given.	LPS	C57BL/6J mice	Improved survival, reduced inflammatory infiltration into lungs and spleen, increased histone acetylation, reduced TNF-α in blood, reduced pro-inflammatory gene expression in lung.
Sodium butyrate	HDAC1 HDAC2	(117)	500 mg/kg (2 doses)	CLP	Wister rat	Improved survival 6 days post-sepsis, protective effect on liver, kidney, and lung
Cambinol	SIRT1 SIRT2	(118)	Prophylactic and therapeutic doses given.	LPS	BALB/c mice	Improved survival, lowered TNFα levels and bacteraemia, blocked phosphorylation of MAPKs.
EX-527	SIRT1	(119)	47 mg/kg	CLP	C57BL/6J mice	Improved survival, reduced TNF-α and IL-6 levels, attenuated bone marrow atrophy.

Histone deacetylases inhibitors targeting classical HDACs are currently used in a number of clinical contexts including cancer. HDACi administration has been shown to attenuate tumor growth and cause apoptosis in tumorigenic cells though the exact mechanism of action is unknown. Synergistic beneficial effects of combinational HDACi use have been demonstrated, although how synergy is achieved is unclear. Vorinostat (or suberoylanilide hydroxamic acid, SAHA) was licensed in 2006 for the treatment of relapsed and/or refractory cutaneous T-cell lymphoma (120). Two other pan-HDACi, for peripheral T-cell lymphoma and multiple myeloma, are also in use (121, 122). Outside of oncology, valproic acid (VPA) is used as an anticonvulsant which acts on class I and II HDACs, and trichostatin A (TSA) is an antifungal which also acts on class I and II HDACs. These examples demonstrate that HDACi treatment, in principle, has an acceptable safety profile, therefore, their use in sepsis is a realistic option should they prove effective.

### Pre-clinical Evidence of HDACi Efficacy in Sepsis

In addition to the positive effects of HDACi on the endothelium during sepsis in mice (discussed above), HDACi treatment has been shown to curb other pro-inflammatory and innate immune responses in pre-clinical models of sepsis. Leoni et al. were the first to report anti-inflammatory properties of Vorinostat both *in vitro* and *in vivo* (123). Prophylactic administration of Vorinostat in mice reduced pro-inflammatory cytokine production upon challenge with LPS. Other reports reveal the impact of VPA and TSA on macrophage activity. Host anti-bacterial responses are inhibited via multiple mechanisms: phagocytic receptors are downregulated and release of reactive oxygen species and nitric oxide is reduced. Bacterial killing, demonstrated in mice with *E. coli* and *S. aureus*, is significantly impeded (123).

Roger et al. demonstrated a significant reduction in mortality in mouse models of toxic shock induced by Pam_3_CSK_4_ and cecal ligation and puncture when HDACi were given (64). TSA negatively regulated the expression of several pattern recognition receptors involved in microbial antigen detection. In addition, they observed that treatment with TSA, Vorinostat, and VPA all repressed cytokine release following TLR stimulation. A recent study exploring the effects of the HDAC6 inhibitor Tubastatin A in a cecal ligation and puncture model of sepsis demonstrated strong therapeutic efficacy. Survival was greater in Tubastatin A-treated mice vs. controls, and pro-inflammatory TNF-α and IL-6 were significantly reduced in peritoneal fluid and plasma of treated animals (106). In addition, a significant reduction in lung injury and bacterial load in the spleen 24 h after cecal ligation and puncture was observed (23 h after HDAC6 inhibitor treatment) (106).

Histone deacetylase inhibitors have been used synergistically with other epigenetic modifiers to ameliorate endothelial integrity and prevent lung injury. Prophylactic inhibition of histone deacetylation alone or combined with inhibition of histone methylation reduced capillary leak and pulmonary oedema in endothelium *in vivo* and substantially minimized lung histopathology (109).

Evidence from the clinic suggests that HDACi could be useful in attenuating the deleterious pro-inflammatory responses seen in sepsis as there is already a precedent for using HDACi in inflammatory disorders. Vorinostat and another HDACi, Givinostat, are licensed therapies for autoimmune inflammatory disorders graft-vs.-host disease and systemic onset juvenile idiopathic arthritis (SOJIA) (juvenile onset Still's disease), respectively (124, 125).

### Considerations and Limitations of HDACi Use

A potential caveat of HDACi treatment is the associated increased risk of subsequent infection that accompanies a reduction in pro-inflammatory responses. Some phase I and II trials of HDACi as cancer treatments noted an increase in severe infections (126, 127) although trials of Vorinostat in graft-vs.-host disease and Givinostat in systemic onset juvenile idiopathic arthritis (SOJIA) have not reported such findings.

It should be noted that the effects of HDACi on the inflammatory response may not be restricted to alterations to the epigenome. The exact effect of HDACi on histone and non-histone acetylation is difficult to characterize, particularly for pan-inhibitors where alterations are likely to be widespread. This impairs our understanding of the exact mechanism driving potentially beneficial effects, in turn hampering the improvement of therapeutic specificity. HDACs have a degree of functional redundancy, therefore knockdown of a given enzyme is frequently compensated for by another of the same class (128). In addition, several HDAC enzymes are known to form multiple complexes, each of which targets a different histone substrate (128).

In several studies of the effects of HDACi on sepsis outcomes treatment was given either prophylactically or very soon following sepsis induction (within an hour), well ahead of the development of symptoms (in CLP models the first symptoms generally appear around 6–12 h post-induction) (Table 1). Therefore, the impact of HDACi treatment in a clinical setting when administered during symptomatic disease is currently unclear.

Histone deacetylase inhibitors may not be appropriate for use in individuals with latent infections. Vorinostat has been proven to reactivate transcription of the HIV reservoir in infected CD4+ T-cells (129). To this end, it has been heavily investigated as part of the “shock and kill” strategy for HIV reservoir eradication (130). Several latent infections such as Epson-Barr virus (EBV) and other herpesviruses also enter lytic replication following HDACi treatment (131). Given the ubiquity of herpesviruses and the seriousness of HIV, reactivation of latent infections during or after a septic episode could be highly detrimental. Whilst HDACi treatment in the context of sepsis is unlikely to be administered long-term, more detailed understanding of HDACi effects on viral latency and reactivation is critical for safe usage.

### The Role of HATs Inhibitors in Inflammation

Given the role of acetylation in sepsis, there is a surprising paucity of data examining the role of HATs activity and inhibition. Whilst less well-characterized than that of HDACs, several studies suggest that HATs inhibition could also elicit anti-inflammatory effects. Several HATs inhibitors including delphinidin, gallic acid, epigallocatechin-3-gallate, diferuloylmethane, and cerulenin have all been shown to reduce pro-inflammatory cytokine release by regulating NF-κB acetylation (132–136). In animal models of acute respiratory distress syndrome and renal injury, elevated HATs activity associated with worsened tissue injury suggesting these inhibitors could have therapeutic benefits in cases of sepsis (137, 138). However, contradictory findings have been reported with some suggesting HATs inhibition has either no effect on pro-inflammatory responses or could in fact exaggerate cytokine release (139, 140). This discordance demonstrates the highly context-specific effect of these drugs. Further, exploration of their role *in vivo* and in sepsis pathophysiology would be welcome.

## Concluding Remarks

Sepsis has a worldwide clinical burden with significant associated morbidity and mortality. Whilst our understanding of the underlying immunopathology has improved over the last 30 years, this has yet to inform effective therapeutic strategies. In this review, we have collated evidence from a large number of studies that highlight the epigenetic mechanisms underlying some of the major aspects of sepsis pathology. Together these reveal the importance of epigenetic changes at transcriptional promotors or enhancers in driving many pathological adaptions. It is key to note that the cell-specific context and stage of sepsis in which these changes occur is important for determining phenotypic effect. The potential use of HDACi as therapeutics in inflammatory disorders has garnered interest over the past decade. These drugs have proven tolerability and are already used in the treatment of a number of cancers. Their mechanism of action is incompletely understood and there are legitimate concerns about off-target effects. Histone deacetylase enzymes are involved in modulating thousands of genes and there are likely to be numerous non-histone targets within the cell that are also affected by their activity. Therefore, detailed exploration of enzyme selectivity and development of more targeted inhibitors are vital next steps in the clinical development of HDACi for use in inflammatory disorders.

We are only just beginning to understand the full scale of epigenetic influence on immune function (141). A critical question to address is the longevity of these adaptions. A limitation of many of the above studies is the relatively short time frame in which epigenetic changes are reported. Results from Mitroulis et al. which demonstrate sustained epigenetic modulation in myeloid progenitors now need to be expanded and built upon (98). A more comprehensive description of both the nature of epigenetic changes and the retention of them is needed to fully understand epigenetic contribution to sepsis pathology and outcome.

## Author Contributions

DC wrote the first draft on the manuscript. RD contributed significantly to the discussion of the clinical presentation of sepsis. RD and DC produced the figures. All authors contributed to manuscript revision, read, and approved the submitted version.

### Conflict of Interest Statement

The authors declare that the research was conducted in the absence of any commercial or financial relationships that could be construed as a potential conflict of interest.
